# Fluctuation structure predicts genome-wide perturbation outcomes

**DOI:** 10.21203/rs.3.rs-7304871/v1

**Published:** 2025-08-12

**Authors:** Benjamin Kuznets-Speck, Leon Schwartz, Hanxiao Sun, Madeline E. Melzer, Nitu Kumari, Benjamin Haley, Ekta Prashnani, Suriyanarayanan Vaikuntanathan, Yogesh Goyal

**Affiliations:** 1 Department of Cell and Developmental Biology, Feinberg School of Medicine, Northwestern University, Chicago IL, USA; 2 Center for Synthetic Biology, Northwestern University, Chicago IL, USA; 3 Robert H. Lurie Comprehensive Cancer Center, Northwestern University, Feinberg School of Medicine, Chicago IL, USA; 4 NSF-Simons National Institute for Theory and Mathematics in Biology, Chicago IL, USA; 5 Department of Ophthalmology, Université de Montréal, Canada; 6 NVIDIA, Santa Clara CA, USA; 7 Department of Chemistry, University of Chicago, Chicago IL, USA; 8 Chan-Zuckerberg Biohub Chicago, LLC, Chicago IL, USA; 9 Data Science of Systems Biology, Technical University of Munich, Germany

**Keywords:** single-cell perturbations, genome-wide responses, linear response theory, fluctuations, Bayesian statistics

## Abstract

Pooled single-cell perturbation screens represent powerful experimental platforms for functional genomics, yet interpreting these rich datasets for meaningful biological conclusions remains challenging. Most current methods fall at one of two extremes: either opaque deep learning models that obscure biological meaning, or simplified frameworks that treat genes as isolated units. As such, these approaches overlook a crucial insight: gene co-fluctuations in unperturbed cellular states can be harnessed to model perturbation responses. Here we present CIPHER (Covariance Inference for Perturbation and High-dimensional Expression Response), a conceptual framework leveraging linear response theory from statistical physics to predict transcriptome-wide perturbation outcomes using gene co-fluctuations in unperturbed cells. We validated CIPHER on synthetic regulatory networks before applying it to 11 large-scale single-cell perturbation datasets covering 4,234 perturbations and over 1.36M cells. CIPHER robustly recapitulated genome-wide responses to single and double perturbations by exploiting baseline gene covariance structure. Importantly, eliminating gene-gene covariances, while retaining gene-intrinsic variances, reduced model performance by 11-fold, demonstrating the rich information stored within baseline fluctuation structures. Moreover, gene-gene correlations transferred successfully across independent studies of the same cell type, revealing stereotypic fluctuation structures. Furthermore, CIPHER outperformed conventional differential expression metrics in identifying true perturbations while providing uncertainty-aware effect size estimates through Bayesian inference. Finally, most genome-wide responses propagated through the covariance matrix along approximately three independent and global gene modules. CIPHER underscores the importance of theoretically-grounded models in capturing complex biological responses, highlighting fundamental design principles encoded in cellular fluctuation patterns.

## INTRODUCTION

Single-cell profiling technologies enable measurements of cell and tissue molecular makeup at an unprecedented resolution and scale^1–3^. Reference collections of the molecular profiles of individual cells across tissues, organisms, and conditions (“atlases”) comprise multimodal measurements, documenting variability within and across cell types through global consortia. Moving beyond the baseline states catalogued by these atlases, recent breakthroughs in experimental platforms, such as Perturb-seq and CROP-seq^4–11^, have enabled the investigation of genetically-perturbed cells by integrating single-cell RNA sequencing with CRISPR screens. Such approaches are transforming functional genomics by providing a systematic, scalable, and high-resolution view of how genetic perturbations drive phenotypic changes. These large-scale perturbation datasets lay the groundwork for mechanistic models that move beyond correlation, toward truly causal maps of gene regulation in single cells^12,13^.

While powerful, high-content pooled single-cell perturbation screens face significant challenges, including few cells per perturbation in addition to the noise and sparsity inherent in single-cell data^2,12,14,15^. These experimental limitations impede reliable detection of genome-wide perturbation effects, especially when using conventional differential expression analyses and significance testing^12,16^. A suite of computational methods have been developed to tackle these limitations^12^. These approaches broadly fall into two categories: interpretable statistical frameworks that analyze existing perturbation data, and predictive models that forecast cellular responses to unseen perturbations. Statistical frameworks include MIMOSCA^6^ and scMAGeCK-LR^17^, which estimate the average impact of each perturbation by fitting linear regression models. Other interpretable methods use matrix factorization (e.g., SVD, NMF, SMAF) to perform dimensionality reduction and identify gene programs or modules that respond coherently to perturbations^18,19^. A notable recent interpretable statistical model is TRADE^16^, which models true differential expression distributions while accounting for noise, using a “transcriptome-wide impact” metric to quantify perturbation effects including weak signals missed by conventional significance testing. At the same time, traditional linear regression models have some limitations: they typically treat genes as independent, cannot explicitly identify the genes driving the response (or the degree of impact per driver gene), and, with the exception of TRADE^16^, produce only point estimates of the regression coefficients and are not uncertainty-aware. Predictive models such as scGen^20^, GEARS^21^, and scGPT^22^, on the other hand, employ deep learning frameworks to model perturbation responses in latent spaces and can predict outcomes of unseen perturbations. These methods often present “black-box” solutions with complex non-linear mappings, limiting interpretability^23^ of the underlying biological mechanisms, and typically require substantial amounts of training data.

Despite progress, we still lack interpretable, physically-grounded approaches with the ability to model genome-wide responses primarily based on the structure of the unperturbed system^24–27^.

**Fig. 1:**
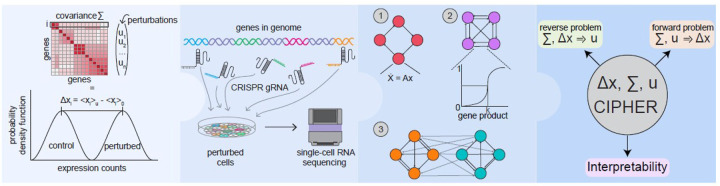
Schematic of the CIPHER workflow and applications to synthetic and real experimental datasets. (column 1) The gene-gene covariance matrix informs on changes to gene expression upon perturbation. (column 2) Perturb-seq interrogates thousands of perturbations by combining RNA-seq with CRISPR screens. (column 3) Linear response can apply to progressively complex synthetic regulatory networks. (column 4) Given covariance and expression changes, CIPHER has three complementary modalities. The forward problem predicts changes in expression, the reverse problem predicts the driving perturbation(s) and the framework is fully interpretable telling us 1) how each genes change is made up of contributions from both itself and every other gene and 2) how response is propagated along dominant or subdominant modes of the covariance.

Nevertheless, techniques from statistical physics help provide a universal framework to predict the effect of perturbations from baseline fluctuations in other contexts^24–27^. If similar physically-grounded models could explain genome-wide perturbation responses, what might that imply about the underlying design principles of biological responses? Here we present CIPHER (Covariance Inference for Perturbation and High-dimensional Expression Response), a framework leveraging linear response theory, which has been foundational in describing complex physical phenomena (Fig. 1)^28–30^. Linear response theory postulates that response to small perturbations is encoded in correlations present within the unforced/unperturbed system’s components. Without necessarily requiring a predefined notion of what constitutes the underlying experimental complexity, linear response theory non-trivially recapitulates experimental findings in a variety of contexts, including the Marcus theory of electron transport and Onsager’s reciprocal relations^28–30^. Here we use CIPHER to identify the determinants of genome-wide responses to genetic perturbations.

We first implemented CIPHER for proof-of-concept recapitulation of responses to perturbations on three classes of synthetic genetic networks where ground truth is known, including stochastic linear and non-linear models of gene regulation. We next applied CIPHER to ten large single-cell perturbation sequencing datasets^16,31–35^ and one imaging Perturb-FISH dataset^10^, including 9 CRISPR interference (CRISPRi) gene knockdown and 2 CRISPR gene activation (CRISPRa) datasets, covering 4,288 perturbations, 1,347,778 cells, and 9 cell types (Table 1). CIPHER robustly recapitulated transcriptome-wide response to single perturbations by using only the information on gene fluctuation covariances in unperturbed (control) single cells. Importantly, eliminating either gene-gene covariances (while retaining individual gene count distributions across cells) in the unperturbed datasets dramatically decreased the model performance. These correlation structures also transferred robustly across independent studies of the same cell type to predict responses. Together, these findings demonstrate the rich information contained within baseline gene-gene fluctuation architectures. Moreover, CIPHER outperformed (AUROC across all perturbations = 0.92) existing metrics for predicting true perturbations given the genome-wide change upon perturbation by leveraging Bayesian inference to measure effect size distributions for all possible perturbations from the correlation matrix. Furthermore, CIPHER enabled decomposition of genome-wide responses into latent regulatory gene programs (“soft modes”), enabling interpretability of our findings. Together, our results on CIPHER highlight the power of simple theoretical models inspired by statistical physics in probing and dissecting biological complexity.

## RESULTS

### Overview of CIPHER

Gene correlations have long served as proxies for gene-gene interactions^36–38^. Recent work reveals these correlations contain meaningful information about gene regulatory network dynamics, particularly during bifurcations^39^. CIPHER builds on these insights, using correlations and a variant of the linear response theory to predict perturbation-induced expression changes. Linear response theory is a classical result in (quasi)equilibrium statistical mechanics which states that the response of a physical system to relatively small external forces is encoded in the correlations of the unforced system. This theory has successfully explained various physical phenomena, including electron transfer kinetics, NMR spectroscopy, dielectric response in polar liquids, and protein conformations and allostery^24–27^. We reasoned that high-content pooled single-cell perturbation screens naturally offer within- and across-gene fluctuations in the unperturbed populations. Moreover, individual gene perturbations can be thought of as “small” given the scale of the system (usually several thousand expressed genes at a given time). Furthermore, as we illustrate below, CIPHER’s success at recapitulating experimental cellular responses suggests a general separation of timescales between fast molecular transcription events and slower state-fate transitions that drive phenotypic changes. This rapid equilibration appears to be a generic feature of gene regulatory and mesoscopic biophysical systems^40,41^.

According to a general formulation of linear response theory, population averages before and after a perturbation vector u is applied are coupled by linear combinations of pre-perturbation gene-gene correlations:

ΔX=Σu

where $\Delta X=\langle X\rangle_{u}-\langle X\rangle_{0}$ and the elements of the covariance matrix Σ are $\Sigma_{ij}=\left\langle \delta X_{i}\delta X_{j}\right\rangle$ for $\delta X_{i}=X_{i}-{\left\langle X_{i}\right\rangle }_{0}$. We denote averages of gene expression X over cells as ⟨X⟩ and distinguish between the perturbed and control ensembles with subscript u and 0 respectively. In a variety of synthetic gene expression networks and experimental Perturb-seq datasets (Fig. 1), we investigated the utility of this framework using a three-pronged approach. First, we considered the forward problem of estimating perturbation responses given true single or double gene perturbations and fluctuation structure Σ. Next, we tackled the reverse problem of predicting perturbations $u=\Sigma^{-1}\Delta X$ from Σ and ΔX. We subsequently examined eigengenes—groups of co-expressed genes that captured the dominant expression patterns—to interpret response as propagating broadly on the gene level but localized to a handful of underlying modes or regulatory modules. Detailed description of CIPHER as well as corresponding derivations and applications are provided in Methods 1.

### CIPHER on synthetic regulatory networks

**Fig. 2:**
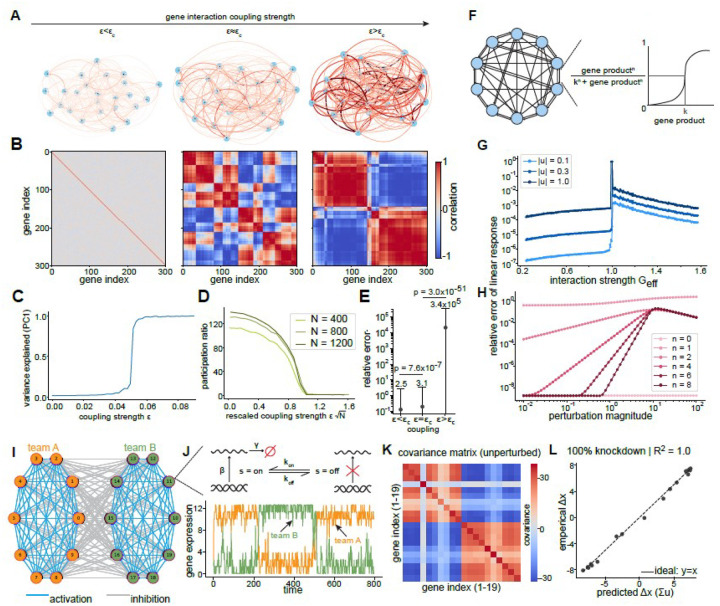
Predicting genome-wide response to perturbations in synthetic regulatory networks. A) Random linear regulatory networks of N genes– nodes represent genes and edge thickness represent the (absolute) strength of interactions between genes. Subcritical, critical and supercritical networks are shown. B) Steady-state gene-gene correlations for the networks in A). C) Percent variance explained by the first principal component as a function of the gene-gene interaction parameter. D) Participation ratio (effective dimension) as a function of the scaled interaction parameter for different sized networks. E) Points are the typical error in linear response, $\|\Delta X-\Sigma u{\|}^{2}/\|\Delta X{\|}^{2}$, across all genes in the three-regimes, averaged over 500 trajectories. Standard error bars shown. (One-sided Wilcoxon signed-rank tests: subcritical error < critical error, p-value = 7.6 × 10^−7^; critical error < supercritical error, p-value = 3.0×10^−51^). F) Non-linear networks with activating Hill function interactions, n = 10. G) Error in linear response as a function of effective interaction strength, G_eff_. Points represent individual simulation runs and lines guide the eye. H) Error in linear response as a function of perturbation magnitude for several different Hill coefficients (n). I) A prototypical ‘teams’ network wherein groups of genes mutually activate within teams and inhibit across them, each gene has its own promoter whose bursting activity is modulated by nonlinear Hill-type reactions with all other genes. J) Time-series of the average expression for the two teams. K) Steady state correlations over the teams time-series. L) Empirical expression change and corresponding linear response predictions. Each point represents an individual gene in the network.

We initially validated CIPHER through proof-of-concept testing on a suite of synthetic gene networks (see Methods 2). These toy networks provided controllable architectures and interaction parameters with known ground truth for benchmarking. We began with linear systems of random gene regulatory networks (dx/dt=Ax+ noise, where A is a matrix of gene-gene interactions and x is a vector of gene expression). We reasoned that CIPHER should accurately recapitulate perturbation responses within the linear regime (Case 1, see Methods 2.1). As Robert May demonstrated in their seminal study^42^, such networks remain stable for N genes when coupling strength $\epsilon_{c}<1/\sqrt{N}$, beyond which they bifurcate to become globally unstable. Consistently, increasing coupling strength (Fig. 2A) caused gene-gene correlations to become modular and fractal-like (Fig. 2B). The transition from the subcritical to supercritical regime is marked by a striking increase in the percent variance explained by the first principal component. Accordingly, a collapse of the dynamics onto a low-dimensional subspace (with low participation ratio) where “team”^43,44^ structure emerges in the covariance as measured by variance explained and participation ratio—a measure of how many components actively contribute to the response (Fig. 2C,D). Biologically, this corresponds to the dynamics passing through an unstable fixed point, as would happen, for example, during a differentiation trajectory^45^. Importantly, CIPHER’s predicted responses to genetic perturbations exhibited relatively low coefficient of variation within the linear regime, but diverged at and beyond the critical coupling threshold (Fig. 2E).

We next tested CIPHER on a prototypical nonlinear dynamical gene expression system consisting of a network of Hill functions (Case 2, Fig. 2F, Methods 2.2). By perturbing a single gene in this nonlinear network, we could directly compare the observed response with CIPHER’s predictions. First, we fixed the Hill-function parameters and varied the gene-gene interaction parameter G, finding a sharp transition at G_eff_ = G/G* = 1 where G* is the critical interaction above which the trivial homogeneous state (zero expression for all genes) becomes unstable (see Methods 2.2 for derivation and Fig. S1A,B for additional examples) (Fig. 2G). Increasing the perturbation magnitude corresponded to a subsequent increase in the relative mean squared error of linear response $\left(\|\Delta X-\Sigma u{\|}^{2}/\|\Delta X{\|}^{2}\right.$, Fig. 2G). We next fixed G and varied the Hill coefficient, n. As expected, CIPHER performed optimally under linear conditions (Hill coefficient, n=0), yielding the lowest error between actual and predicted values (Fig. 2H). However, as nonlinearity increased (Hill coefficient >1), error rates peaked during transition periods but remained low otherwise—a pattern consistent with the characteristic response curves of Hill function dynamics (Fig. 2H). We hypothesized that CIPHER’s prediction accuracy should decline under more challenging perturbation conditions. Specifically, we tested whether increasing either the perturbation magnitude $\left(u_{i}\right)$ for single gene knockouts or the total number of simultaneously perturbed genes would degrade performance. Indeed, CIPHER performance systematically decreased as we increased both the magnitude (u_i_) and total number of simultaneous perturbations (u_i_, where I ∈ {1,2,3,4,5}) (Fig. 2F–H and S1C), confirming that the method’s linear assumptions become limiting under extreme perturbation conditions.

Lastly, we tested CIPHER on a stochastic non-linear network capturing the recently described “teams” topology^43,44^, where genes on the same team mutually activate each other and there is inhibition between genes on different teams (Case 3, Fig. 2I, see Methods 2.3 for details on the model construction). Teams can emerge within gene regulatory networks and have been implicated in driving phenotypic transitions (e.g., epithelial-mesenchymal transition) in multiple cancers and during development^43,44^. Interestingly, despite the heterogeneity in the interaction parameters and a sparse interaction network, the synthetic system functions as a collective bi-stable switch toggling stochastically between two fixed states (high A, low B, and conversely) (Fig. 2I,J). We found that CIPHER readily captured $\left(R^{2}=1-\|\Delta X-\Sigma u{\|}^{2}/\|\Delta X{\|}^{2}=1.0\right.$) the responses to perturbations (‘knocking out’ a gene by setting its individual gene expression to zero) in this nonlinear stochastic model, which incorporates transcriptional bursting and a Hill function response (Fig. 2K,L). Collectively, our proof-of-concept implementation of CIPHER on multiple toy synthetic gene networks demonstrates its versatility across diverse dynamical systems under controlled conditions.

### CIPHER captures genome-wide perturbation responses in real datasets

**Fig. 3:**
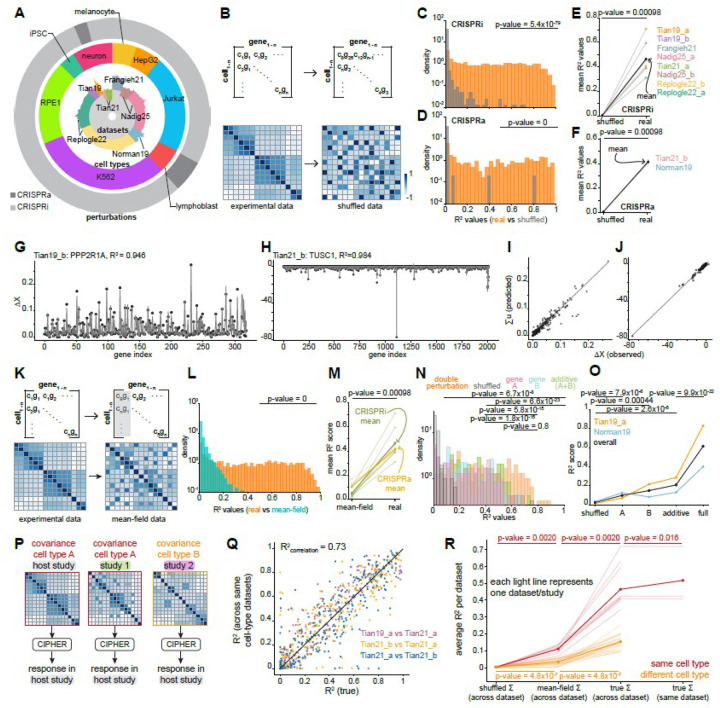
The forward problem: to what extent can covariance structure explain response to known gene perturbations from Perturb-seq. A) We consider 10 Perturb-seq datasets, comprising CRISPRi (8 datasets) and CRISPRa (2 datasets) gene perturbations (outermost ring) for 8 different cell types (middle ring). The innermost ring shows the log-number of control (grey) and perturbed (colored) samples for each perturbation in each dataset. B) Show control gene expression matrices before and after shuffling rows and columns, as well as corresponding covariance matrices C) Distributions of $R^{2}$ values over all CRISPRi perturbations, optimizing over the known perturbation with either the real or shuffled covariance matrix (KS test stat = 0.881, p-value = 5.444×10^−79^). D) Distributions of $R^{2}$ values over all CRISPRa perturbations, with real or shuffled covariance (KS test stat = 0.876, p-value = 0). E) Average $R^{2}$ values over all CRISPRi perturbations (black points, black line) and per dataset (colored points, grey lines) F) and analogously for CRISPRa (p-value calculated using combined one-sided Wilcoxon signed-rank tests including all 10 datasets: p-value = 0.00098). G) The top scoring perturbation for Tian_19b is PPP2R1A. We show the empirical ΔX (lines) as well as that predicted by linear response (dots). H) The top scoring perturbation for Tian_21b is TUSC1. I) Predicted ΔX=Σu vs experimental data for PPP2R1A, each point is a gene, J) and similarly for TUSC1. K) Real and ‘mean-field’ (shuffled over cells, not genes) gene expression count matrices, and corresponding covariance matrices. L) Distribution of $R^{2}$ values over all datasets for mean-field and real covariance matrices (KS test stat = 0.654, p-value = 0). M) The average $R^{2}$ values for CRISPRi/a datasets with the real Σ compared to mean field. Dark points are averages over all CRISPRi (green) and CRISPRa (yellow) perturbations, and light points are per dataset averages. (Combined one-sided Wilcoxon signed-rank tests including all 10 datasets: p-value = 0.00098) N) Histograms of $R^{2}$ values over all double perturbations to different genes A and B. We compare the full double perturbation response to that achieved for shuffled X_0_, perturbations A or B alone, and the additive solution. (Shuffled vs Single A: KS stat = 0.5250, p-value = 1.823×10^−15^; Shuffled vs Single B: KS stat = 0.5167, p-value = 5.753×10^−15^; Shuffled vs Additive: KS stat = 0.6333, p = 6.627×10^−23^; Single A vs Single B: KS stat = 0.0833, p-value = 8.012×10^−1^; Additive vs True Σ: KS stat = 0.3750, p-value = 6.651×10^−08^). O) Mean $R^{2}$ across the conditions in N) (Shuffled vs Single A: p-value = 7.9×10^−6^; Shuffled vs Single B: p = 0.00044; Shuffled vs Additive: p-value = 2.6×10^−6^; Additive vs True Σ: p-value = 9.9×10^−22^). Orange points are over Norman19 perturbations, blue points over Tian_19a perturbations and the black points are the averages over both the datasets. P) Responses in a host study from inter-study covariances either of the same or different cell type. Q) $R^{2}$ values across neuron datasets. $R^{2}$ values using the true covariance matrix from the host study compared to those from a different dataset but same cell type. Each point is a perturbed gene. R) Average $R^{2}$ values across all pairs of datasets and conditions, using a covariance matrix from either a shuffled X_0_ (across datasets), the mean-field approximation (across datasets), the unshuffled X_0_ (across datasets) and the unshuffled X_0_ from the same dataset. Thin lines correspond to individual dataset pairs (averages over all perturbations in the host dataset with a Σ of the same cell type in red or different cell types in orange) and points are averages over the typical $R^{2}$ values for these pairs of datasets. Note that there are only three lines connecting the across and within dataset real covariance for the same cell type. This is because we are comparing the three datasets shown in Q, and that there is no true covariance from the same dataset and different cell type. (Same cell type Shuffled vs. mean-field: p = 0.0020; Same cell type mean-field vs cross-dataset covariance: p-value = 0.0020; Same cell type cross-dataset covariance vs same dataset covariance: p-value = 0.016. Different cell type Shuffled vs. mean-field: p-value = 4.8×10^−7^; Different cell type mean-field vs true: p-value=4.8×10^−7^).

Complex gene regulatory networks can be viewed as out-of-equilibrium systems with underlying correlations between genes. We questioned whether the CIPHER framework might recapitulate genome-wide responses to perturbations, which are typically a single gene knockdown or activation at a time. To address this question, we first systematically collected and performed quality control on existing single-cell perturbation datasets (see Methods 3,6). In total, we analyzed ten high-throughput perturbation datasets, containing 8 CRISPR interference gene knockdown and 2 CRISPR gene activation datasets and covering 4,234 perturbations, 1,342,852 cells, and 8 cell types (Fig. 3A, Table 1). We calculated covariance matrices for unperturbed single cells from each experimental gene count matrix (raw absolute counts for each gene) and from corresponding scrambled control matrices which constitute a null model (Fig. 3B). We then implemented CIPHER on the forward problem of predicting transcriptome-wide response, optimizing for a single u_i_* corresponding to the true perturbation i of interest and measuring the response by calculating an R^2^ value (Fig. 3C,D). An R^2^ = 1 implies that we could perfectly transport the initial vector of gene expression $\langle X\rangle_{0}$ to the final measured target $\langle X\rangle_{u}$. Remarkably, we found that CIPHER alone was able to significantly recapitulate the transcriptome-wide responses as compared to the null model for both activating and interfering perturbations, with some R^2^ values approaching 1 (Fig. 3E,F). We have illustrated gene-wise concordance between CIPHER predictions and experimental data for a few representative test cases in Fig. 3G–J and S2. We explored the mechanistic underpinnings of variability in R^2^ values across datasets later in the results section. We note, however, that the per-gene values of R^2^ had little dependence on the control cell expression (Fig. S3A).

We next reasoned that if the co-fluctuations between the genes in the unperturbed population were indeed critical to the CIPHER framework, simply shuffling the gene counts column-wise—which retains the original mean and distribution for each gene individually but scrambles the covariance—should result in reduced R^2^ values (Fig. 3K). This kind of shuffling operation, equivalent to a widely used ‘mean-field approximation’ in physical systems, indeed resulted in reduced R^2^ values (Fig. 3L, M and S3B), confirming the dominant role of off-diagonal elements of the covariance matrix in predominantly driving transcriptome-wide response to activating and repressive genetic perturbations. Even optimization for any random gene u* in this mean-field model did not improve the R^2^ values (Fig. S3C), further consolidating the dominant role of the gene-gene covariances in driving transcriptome-wide outcomes. We also implemented CIPHER on recently proposed optical screens like Perturb-FISH^10^, with similar high-confidence results (Fig. S3D).

### CIPHER captures response to double perturbations

Pooled single-cell CRISPR screens provide rich datasets for investigating epistasis and gene interactions in cases where the same cell receives guides perturbing multiple genes. We implemented CIPHER to evaluate the relationships between the genes perturbed and the respective outcomes. For each double perturbation (2 genes perturbed simultaneously), we performed a similar optimization, but in this case over $u_{i}$ and $u_{j}$ instead of just a single $u_{i}$. This optimization contains a nonlinear cross term that couples $u_{i}$ and $u_{j}$ through the off-diagonal element of the covariance matrix $\Sigma_{ij}$, so that epistasis manifests itself in this model directly through $\Sigma_{ij}$. When tested on two datasets with double perturbations (Table 1), we found that the full model outperformed the additive solution $u_{i}+u_{j}$ in a vast majority of cases (Fig. 3N,O, S3E). Biologically, these results suggest that many true double perturbations are to some extent epistatic. Moreover, as was the case for synthetic networks, as the number of simultaneous perturbations per cell gets larger, the additive performance decreases (Fig. S3F). Notably, the additive model performs better overall than the baseline null model that predicts multi-gene perturbation response as the average of individual gene responses (Fig. S3F). Our results also highlight how CIPHER can be leveraged to discriminate between additive or epistatic relationships.

### Predictive power of covariances are preserved across studies of same cell type

We tested whether the gene-gene co-fluctuations in unperturbed cells from one dataset or experiment can be informative in predicting perturbation response in another independent study of the same cell type or cell line. If so, it would suggest a universal underlying structure of covariance that carries essential information for predicting responses to perturbations. Conversely, gene-gene covariances in each experiment—regardless of whether cell type is shared—are sensitive to specific experimental conditions. To test this idea, we focused on three unrelated datasets with a shared cell type (neuron) (Table 1, Fig. 3A). We calculated covariances from two of the datasets and used CIPHER to predict responses in the third “host” dataset (Tian21_a, Fig. 3P). Remarkably, we found strong correlation (R^2^=0.73) between transcriptome-wide responses calculated from cross-dataset covariance matrices of the same cell type (Fig. 3Q) and typically much poorer performance from cross-dataset of a different cell type (Fig. S3G). Similar analysis across distinct cell types/lines failed to recapitulate transcriptome-wide responses (Fig. 3R). However, real covariances of unrelated cell types outperformed their corresponding mean-field models (shuffling the gene counts column-wise, which retains the original mean and distribution for each gene individually), suggesting the presence of certain universal, albeit weakly-contributing, fluctuation structures that inform outcomes (Fig. 3R). To test whether cross-experiment covariance matrices retain predictive ability in primary tissue beyond the single-cell perturbation datasets, we analyzed three single-cell atlases from CellxGene^46^: human embryonic meninges (5–13 weeks post conception, 58 cell types), human intestine organoid (22 cell types), and human lung organoid (9 cell types)^47,48^. The meninges dataset contained many neuron-related cell types, while lung and intestine datasets both predominantly contained non-neuron cell types. Most cell types in the embryonic meninges dataset better recapitulated cell culture perturbation responses than their intestine and lung counterparts, with the only neuron cell type in the intestine dataset performing best among all intestine cell types (Figure S3H–K). These findings are consistent with a recent study outlining the presence of shared and cell-type-specific correlative gene modules between the human osteosarcoma (U2OS) and human embryonic kidney (HEK293T) cell lines^49^. Collectively, these results suggest the presence of cell-type specific strongly-contributing and universal weakly-contributing latent fluctuation structures which can be harnessed to predict transcriptome-wide responses.

Inspired by the fact that cross-experiment covariance matrices from neurons to large extent retain the ability to predict perturbation response in cell culture, we ventured to ask whether this holds true for primary tissue as well. To test this, we analyzed three single cell atlases from CellxGene: 1) human embryonic meninges at 5–13 weeks post conception, 2) human intestine organoid, and 3) human lung organoid. These datasets had 58, 22, and 9 labeled cell types, respectively. Of the three datasets, the first contains many neuron-related cell types and the first two contain neurons. Testing how well perturbation response in cell culture is predicted by these primary cells, we found that most cell types in the embryonic meninges dataset recapitulated response better than their counterparts in intestine and lung organoids. However, we note that the neuron cell type in the intestine did the best at predicting response of all the intestine cell types. To compute the covariance matrices, we used as many cells as there were neurons in the meninges dataset.

### CIPHER accurately predicts true perturbations

**Fig. 4:**
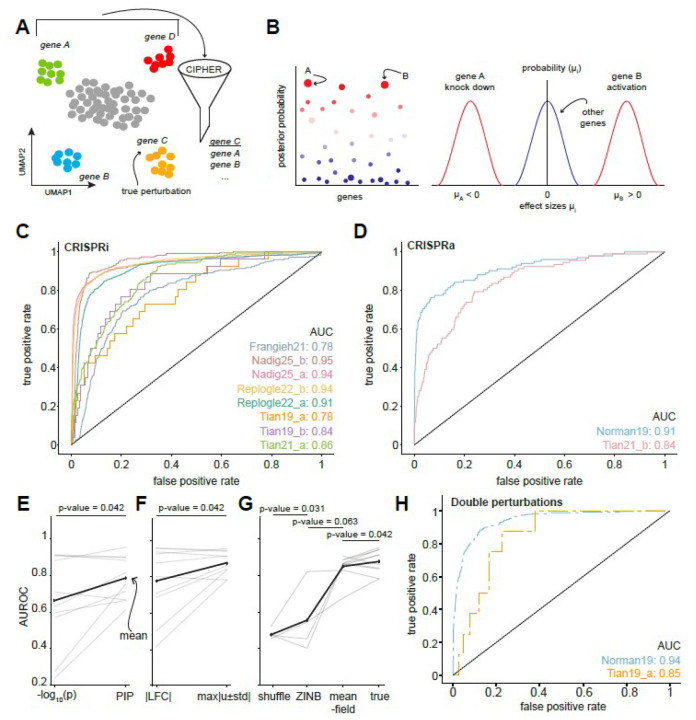
The inverse problem: predicting causal drivers of perturbation response in transcriptome-wide screens. A) CIPHER ranks genes responsible for a measured perturbation using knowledge of the control-cell gene expression fluctuations. Each point corresponds to a transcriptome from a single cell. B) Left: Posterior probability of a nonzero perturbation effect across genes (points). True perturbed genes A and B have high posterior probabilities. Right: effect size distributions for different perturbations. Distributions of effect size for genes A and B as well as a composite distribution over other unperturbed genes. C) Receiver-operator characteristic curves for each CRISPRi dataset. Each curve corresponds to a dataset. D) Receiver-operator characteristic curves for each CRISPRa dataset. E) AUROC comparison across metrics: p-value vs. PIP. (One-sided Wilcoxon signed-rank test p-value = 0.042) F) AUROC comparison across metrics: log fold change vs maximum posterior effect size distribution spread vs. PIP. Each line connects AUROC scores for the same dataset over different conditions. (One-sided Wilcoxon signed-rank test p-value = 0.042) G) AUROC comparison across covariance conditions: shuffled, ZINB, mean-field and the true covariance. Note that ZINB only has 4 datasets associated with it, as reparameterization for the other datasets led to pathological results (negative mean, see Methods 4). (Shuffled Σ vs ZINB (n=4): one-sided p-value = 0.31; ZINB vs Meanfield (shuffled X_0_) (n=4): one-sided p-value = 0.063; Meanfield (shuffled X_0_) vs Real Σ(n=10): one-sided p-value = 0.042) H) Receiver-operator characteristic curves across datasets containing double (2-gene) perturbations.

Having tested CIPHER in the forward direction on capturing genome-wide response to single and double perturbations, we next investigated the inverse problem: given a covariance matrix and a transcriptome-wide change in gene expression, what gene (or genes) most likely induced the change (Fig. 4A)? To address this question, we solved for a full combinatorial gene perturbation u (a potentially nonzero element $u_{i}$ for all genes) as $\Sigma^{-1}\Delta X$. We argued that ordering the resulting $u_{i}$ by magnitude should, in principle, provide an effective ranking of $\text{gen}e_{i}$ and a point estimate of whether they were likely knocked-down or activated. High-throughput Perturb-seq data provides an excellent platform to solve this inverse problem since the ground-truth perturbations are known *a priori*. To incorporate the possibility of error in the estimates of the averages, covariances, or deviations from linearity, we reframed the question as a Bayesian linear regression problem (Fig. 4B). Concretely, we sample from the posterior distribution $p\left(u_{i}|\Delta X,\Sigma \right)$ assuming some prior knowledge on sparsity (i.e., only a few gene perturbations should have significant nonzero effects). This is accomplished by introducing a prior distribution over the perturbations p(u) which has support over zero (no effect) and a mode away from zero occupied by true perturbation effects (see Methods 4). This operation enabled us to put error bars on estimates of perturbation effects, and assign each gene a posterior inclusion probability (PIP) that it has true nonzero effect (Fig. 4B, S4A). Together, the Bayesian approach allowed CIPHER to provide uncertainty-aware estimates of the perturbation effect sizes that the naive point estimates could not.

To make the problem tractable, we performed Markov-Chain Monte Carlo on the top 200 prospective perturbations according to $\Sigma^{-1}\Delta X$, forcibly including the true perturbation if needed (see Methods 4). We found that often when the true perturbation is originally ranked low by the naive point estimate, it rises through the ranks during inference to become one of the, if not the most, highly probable genes with the largest magnitude effect size of the top_k_ (median rank increase: 28.2, see Fig. S4B).

For each dataset in Table 1, we compared the ranking metrics ($\text{PIP},\text{max}\left(<u_{i}>+/-\sigma_{i}\right)$) with receiver-operator characteristic (ROC) curves, finding that ranking the perturbations by the maximum spread $\text{max}\left(<u_{i}>+/-\sigma_{i}\right)$ proved most informative of the true perturbation (Fig.4C–G). We found that regardless of the dataset, cell type, or perturbation type (CRISPRi or CRISPRa), CIPHER achieved high values on the receiver-operator characteristic curves for maximum spread and PIP (Fig. 4C–E). Next, we wondered how CIPHER results compare to conventional metrics used in differential analysis of control and perturbation conditions, such as log fold-change (logFC) and associated p-values. We found that CIPHER outperformed these conventional metrics in identifying the true perturbation, particularly for datasets with relatively lower mean AUROC values (Fig. 4E–G). CIPHER exhibited high performance for double perturbations datasets as well (Fig. 4H). Unlike conventional metrics, CIPHER calculates how much each gene contributes to other genes’ expression changes, powering it to reveal the mechanistic underpinnings of cellular response, which we cover in the next section.

### Soft modes drive genome-wide responses to perturbations

Given CIPHER’s success in capturing transcriptome-wide responses, we next examined the underlying drivers of its high performance. CIPHER inherently quantifies how each gene influences every other gene’s expression, in principle allowing us to interpret both gene- and “regulatory module”-level contributions to responses. We first investigated the dimensionality of the response. That is, to what extent the transcriptome-wide response is propagated along specific directions or “soft modes” of the covariance matrix. (A soft mode is a direction of change that the system can move relatively easily along and mathematically corresponds to a dominant principal component.) We decomposed the covariance matrix into its eigen-representation, where eigengenes constitute effective regulatory modules of multiple co-expressed genes, each capturing a different amount of variance. Should the variance captured by a given eigengene dominate over others, it is considered a soft mode. We confirmed the presence of soft modes in the datasets, as revealed by the high magnitude of eigenvalues of the covariance matrix (Fig. S5A). Such soft modes have recently been argued to be ubiquitous in transducing response to perturbation in complex biophysical systems, illustrating the utility of adopting a linear response framework like the one employed in our study.^50^

**Fig. 5:**
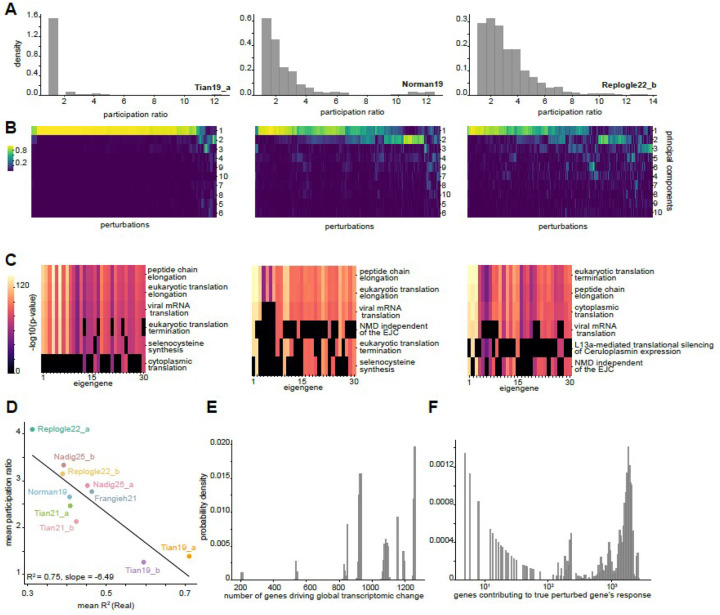
The effective dimensions of transcriptome-wide response. A) Distributions of participation ratio (effective dimension) across all perturbations for three Perturb-seq datasets. B) Clustered heatmaps of the fraction of the response that falls along each principal component for the datasets in A). C) Gene-ontology significance heatmaps (clustered) from gene sets with high-loadings in each principal component. D) Mean participation ratios vs. mean $R^{2}$ values over the CRISPRi/a Perturb-seq datasets considered. Each point corresponds to a dataset and we show the best fit line describing the linear trend. E) The effective number of genes driving global change, calculated from the entropy of the fractional contributions of each gene’s change from every other gene. F) The effective number of genes impacting the expression change of the true perturbed gene across datasets, calculated in the same way as E).

For each perturbation in each dataset, we additionally calculated a participation ratio. The participation ratio—a measure of how many eigengenes actively contribute to the response—of a vector is a measure of how many nonzero elements it has: it evaluates to 1 when there is one nonzero and to the length of the vector when all elements have equal size. Specifically, we evaluated the participation ratio of the squared overlap (dot product) of each eigengene with the total response vector ΔX, yielding an estimate of the effective dimensionality of response. We reasoned that a low participation ratio could contribute to higher CIPHER performance. Indeed, the participation ratio was generally low relative to the dimension of the datasets (mean = 3.07 eigengenes over all perturbations) with notable variation between datasets (Fig. 5A and S5B–D). Interestingly, a majority of responses in Tian19_a (Table 1)—which exhibited the highest R^2^ values (Fig. 3E)—could essentially be described with a single dimension. Conversely, datasets with relatively high participation ratios correspondingly exhibited lower R^2^ values (correlation R^2^ = 0.75; slope = −6.5) (Fig. 5D). We observed consistent results when comparing the normalized contribution of each eigengene for each perturbation, again illustrating that Tian19_a responses were typically well described by their first principal component, unlike the other datasets (Fig. 5B). Next, we checked whether certain gene programs underlie dominant eigenmodes by calculating the dominant genes from the eigengene loadings for each principal component, and performed a gene ontology^51^ search on them. In line with a recent study on covariances in mammalian cells^49^, the majority of processes are related to translation and general cellular homeostasis (Fig. 5C), a trend that holds true over all datasets combined as well as within datasets from a single cell type (Neuron, Fig. S5F,G). This structure reflects an intrinsic coordination essential for maintaining cellular homeostasis—specifically, at and near equilibrium states, cellular processes perhaps prioritize general growth pathways; these pathways may serve as the primary conduits through which perturbations are transduced in cells.

### CIPHER provides estimates on total genes affected during perturbations

We next sought to calculate the effective number of differentially expressed genes upon perturbation. CIPHER provides the total normalized contribution to the expression change of $\text{gen}e_{i}$ due to correlations with $\text{gen}e_{j}$, and the analogous contribution of each gene to the total response. We calculated the entropy of these two probability vectors and exponentiated it to obtain an effective number of genes responsible for two kinds of estimates: 1) the expression change in the true perturbed gene, and 2) the total transcriptomic change upon perturbation (see Methods 5.3). Entropy-based estimates suggest that the transcriptomic response is distributed across a large number of genes (Fig. 5E, S5D), consistent with prior findings^16^ that essential gene perturbations trigger widespread but diffuse expression changes. Many perturbed genes showed high self-variance contributions to their expression changes, implying that the perturbations directly affected the gene being targeted, as expected (Fig. S5E). Furthermore, while specific gene interactions could significantly affect perturbation responses, most essential gene perturbation effects appeared to emerge from many smaller correlations between the perturbed gene and its partners (Fig. 5F). Collectively, our results highlight despite the presence of soft modes, each soft mode represents a complex, yet quantifiable milieu of gene interactions.

### Benchmarking CIPHER predictive power and computational costs

We compared CIPHER performance and computational efficiency against other existing analytical and deep learning models. We benchmarked CIPHER against another linear model, MIMOSCA^6^, as well as two deep learning approaches: a CPA-like autoencoder (adapted from the original CPA framework designed for drug perturbations^52^) and GEARS^21^ (see Methods 7). We used two kinds of R^2^ scores: ${\text{R}}^{2}(\Delta \text{X})=1-{\left\|\Delta X-\Delta X^{\prime }\right\|}^{2}/\|\Delta X{\|}^{2}$ and $R^{2}\left(X_{1}\right)=1-{\left\|X_{1}-X_{1}\prime \right\|}^{2}/{\left\|X_{1}\right\|}^{2}$. Both scores quantify how well perturbation effects are captured, though ${\text{R}}^{2}(\Delta \text{X})$ by measuring goodness of fit relative to a null prediction of X_0_ (control expression) rather than zero expression. All methods achieved high R^2^(X_1_) scores, with median values of: CIPHER = 0.989, MIMOSCA = 0.975, CPA-like autoencoder = 0.982, GEARS with 64 neurons and 20 connections = 0.996, and GEARS with 16 neurons and 5 connections = 0.940 (Fig. S6A,B). However, when comparing ${\text{R}}^{2}(\Delta \text{X})$ values, methods other than CIPHER yielded negative scores for a subset of perturbations, indicating that perturbation responses predicted by the other three approaches were often further from true responses than simply predicting the average control vector (Fig. S6C,D). Notably, the performance of GEARS, with its extensive parameter set, proved sensitive to both model architecture and training conditions for both R^2^(X_1_) and ${\text{R}}^{2}(\Delta \text{X})$ metrics (Fig. S6C,D). While both deep learning models and the linear MIMOSCA model contain approximately one million free parameters, CIPHER requires fitting only a single parameter to determine the optimal correlation scale (Fig. S6E). Finally, CIPHER’s total computational resource requirements—combining runtime and memory usage—were orders of magnitude lower than any other method tested (Fig. S6F,G), highlighting the predictive power and low computational costs of CIPHER.

## DISCUSSION

Here, we developed CIPHER, a framework combining linear response theory, Bayesian statistics, and regulatory networks to model rich single-cell perturbation experiment outcomes. We benchmarked CIPHER on synthetic networks, demonstrating its capability to predict systemic responses to perturbations. Applied to experimental single-cell perturbation datasets (Table 1), CIPHER reliably captured transcriptome-wide responses to gene perturbations—an effect that disappeared when gene-gene fluctuations in unperturbed cells were eliminated. Moreover, gene-gene fluctuations from one dataset performed similarly well in other datasets of the same cell type, highlighting the presence of stereotypic genome-wide fluctuation structures within cell types. CIPHER also identified the true perturbation with high-fidelity given only the covariance matrix and change in gene expression upon perturbation, while also revealing that transcriptome-wide responses tend to align with soft eigenmodes. Each soft mode captures a quantifiable ensemble of weakly- and strongly-coupled gene interactions that confer predictability to responses. In sum, our results demonstrate that theoretical approaches drawn from statistical physics like CIPHER offer powerful tools for fundamentally understanding the responses to a large class of perturbations, despite the underlying biological complexity.

CIPHER’s success in predicting cellular responses reflects the broader power of the linear response theory, which has proven remarkably effective across a diverse array of complex systems. In physical sciences, linear response theory underpins foundational theories like Marcus electron transport theory and Kubo’s Fluctuation-Dissipation theorem in non-equilibrium statistical mechanics, explaining phenomena ranging from NMR spectroscopy and dielectric response to protein allostery^24–27^. CIPHER’s ability to accurately predict complex, transcriptome-wide responses using a simple, interpretable linear model suggests that biological systems may operate in regimes where linear response theory remains surprisingly effective despite underlying nonlinear dynamics. The presence of coordinated fluctuations and their important contribution to responses to genetic and environmental perturbations has been reported for diverse microbial and mammalian behaviors across scales including transcriptional, protein, and metabolic cell states^49,53–55^. In fact, the authors of one particular experimental study found steady-state single-cell gene correlations could predict the effect of p53 perturbation more accurately than even chromatin immunoprecipitation studies^49^. Yet, these observations came from the perturbed gene correlations, and had not been explicitly rationalized or quantified within a theoretical framework. As such, our study provides a strong theoretical footing toward a fundamental biological principle: complexity can emerge from or be effectively captured by simpler mechanistic rules encoded in the fluctuations of *unperturbed* cellular states. Such fundamental insights offer a path toward mechanistic rather than purely correlative or black-box understanding cellular responses to perturbations or lack thereof^56^.

Given our results within and across cell-types, in principle, CIPHER could be harnessed to reveal the hierarchical relationships between cell types on underlying genome-wide fluctuation structures. Going further, we imagine CIPHER could be employed across species to probe the evolutionary history of gene-gene correlation. In its present form, CIPHER takes each dataset as input separately. Future studies could couple the CIPHER framework with existing analytical and deep neural network approaches^52,57–62^ for joint embeddings of disparate datasets. Furthermore, incorporating a CIPHER-based consistency loss that leverages pre-computed gene-gene covariance matrices could enhance the generative modeling of perturbation effects. By encouraging the model to preserve these covariance structures, this approach may improve generalization to unseen cell types and perturbations.

CIPHER demonstrated that transcriptome-wide perturbation effects can emerge through different modes: concentrated effects from a small subset of highly responsive genes (u_i_) versus distributed effects where many genes contribute modestly but cumulatively significant changes. These findings, combined with effect size calculations and comparison from TRADE^16^, point to the potential application of CIPHER in designing efficient attention or, even, tokenization mechanisms^63,64^ to enhance the predictive power of existing deep learning models or designing newer, more expressive, cellular foundation models. While CIPHER effectively captures responses in many cases, it sometimes fails due to nonlinearities and factors beyond direct gene-gene fluctuations, as well as higher response complexity in some datasets. Incorporating the recently proposed single-cell clone tracing technologies^2,65–67^ with pooled CRISPR screening could enhance the accuracy of CIPHER by accounting for clone-specific fluctuations.

In a similar spirit to CIPHER, spin-based, Ising-type models from statistical physics have recently been adapted to infer effective gene regulatory networks from single-cell RNA sequencing data and to predict cellular responses to perturbations^68^. Looking ahead, we anticipate the emergence of technologies capable of capturing time-resolved single-cell perturbation dynamics, which would demand new theoretical frameworks. Response theories for (non)linear time-dependent perturbations^69^, fluctuation theorems^70,71^, and universal thermodynamic bounds and control theories^72–75^ are all derived from the recently developed nonequilibrium statistical physics of entire trajectories, and could be adapted to open new frontiers in the quantitative understanding of cellular regulation through the lens of nonequilibrium fluctuations.

Although beyond the scope of the current work, CIPHER could be harnessed to predict unknown perturbations driving transitions in complex disease traits such as cancer metastasis and drug resistance to targeted therapy^76^, genetic disorders with unknown causal genes^77^, and more, significantly shortening the list of possible causal drivers. In such cases for example, one would compute the covariance of the naive cancer cells before drug as well as the response to drug and apply the inverse problem as we have formulated it here. This could constitute a standalone procedure without necessarily the need for integration of prior perturbation data, as demonstrated by other recent approaches combining trait and perturbation datasets^77^. Scaling up perturbation experiments with AI and statistical physics could enable efficient virtual screens to map genotype to phenotype beyond current single-trait CRISPR studies.

## Methods for CIPHER

Here we detail the methods and procedures for CIPHER. We start by deriving the results of linear response theory and motivating its use in describing transcriptional changes. Next, we discuss simulations for the synthetic regulatory networks considered in the main text. From here, we consider the ‘forward’ and ‘inverse’ problems in turn, and conclude by describing the analysis surrounding our discussion of soft modes in propagating the response of gene expression to cellular perturbation.

### 1 The case for transcriptome-wide linear response theory

We now describe the theoretical foundations of CIPHER. Linear response is a classical result in (quasi)equilibrium statistical mechanics [1, 2] which states that the typical response of a physical system to some relatively small external force is encoded in the correlations of the unforced system. Denoting the state of the unperturbed system as $x=\left(x_{1},x_{2},\ldots \right)$, the force as u and the covariance as Σ so that $\Sigma_{ij}=cov\left(x_{i},x_{j}\right)$, the main result of static linear response is

(1) $$\langle x\rangle_{u}=\langle x\rangle_{0}+\Sigma u.$$

or

(2) $$\left\langle \Delta x_{i}\right\rangle =\sum_{j}{\left\langle \delta x_{i}\delta x_{j}\right\rangle }_{0}u_{j}.$$

This result naturally emerges from many realistic physical contexts. Namely, Eq. 2 holds: 1) for any quasi-stationary system where the perturbation u is *conjugate* to the expression state variable x 2) in a number of simple transcriptional bursting models, and 3) in systems with soft modes that dominate the spectrum of the covariance. We discuss each of these scenarios below.

#### 1.1 Quasi-stationary systems with X conjugate to u

We consider a system in a steady state described by a probability distribution $p_{0}(x)$, where $x\in R^{N}$ is the state vector. The system is perturbed by a small external field $u\in R^{N}$, such that the perturbed distribution becomes:

(3) $$p_{u}(x)=\frac{1}{Z(u)}p_{0}(x)e^{u^{⊤}x}.$$

This form implies that x is the sufficient statistic and is conjugate to u in the exponential family sense. The normalization factor Z(u) is the moment generating function:

(4) $$Z\left(u\right)=\int dxp_{0}\left(x\right)e^{u^{⊤}x}.$$

The expectation of x under the perturbed distribution is

(5) $$\langle x\rangle_{u}=\int dx\;xp_{u}(x)=\frac{1}{Z\left(u\right)}\int dx\;xp_{0}(x)e^{u^{⊤}x}$$

which can be written as the gradient of logZ(u),

(6) $$\langle x\rangle_{u}=\nabla_{u}\text{log}\;Z(u).$$

Now taking the derivative of $\langle x\rangle_{u}$ with respect to u to obtain the response

(7) $$\frac{d}{du}\langle x\rangle_{u}=\nabla_{u}\nabla_{u}^{⊤\;}\text{log}\;Z(u)={\text{Cov}}_{u}(x).$$

Evaluating this at u=0, we recover the covariance matrix of the unperturbed system,

(8) $${\left.\frac{d}{du}\langle x\rangle_{u}\right|}_{u=0}=\Sigma ,\;\text{where}\;\Sigma_{ij}={\left\langle x_{i}x_{j}\right\rangle }_{0}-{\left\langle x_{i}\right\rangle }_{0}{\left\langle x_{j}\right\rangle }_{0}.$$

Therefore, to leading order in u, the response of the mean is

(9) $$\Delta \langle x\rangle =\langle x\rangle_{u}-\langle x\rangle_{0}=\Sigma u+𝒪\left(\|u{\|}^{2}\right).$$

This is the static linear response formula for systems where the perturbation enters the distribution as $e^{u^{⊤}x}$, and is valid under the assumption of quasi-equilibrium (i.e., fast microscopic relaxation compared to the timescale of u).

#### 1.2 Heuristic examples showing the perturbation is conjugate to gene expression

In the following examples we write the mean equations of motion and assume unless otherwise stated that gaussian white noise determines fluctuations around these mean equations, i.e. that the noise amplitude $\left\langle \eta_{t}^{2}\right\rangle =2D$.

##### 1.2.1 Example 1: Constant-rate transcription with linear perturbation

Consider a gene with linear degradation and production perturbed by a small additive input u

(10) $$\frac{dx}{dt}=\beta +u-\gamma x.$$

At steady state (dx/dt=0), the solution is

(11) $$x^{*}=\frac{\beta +u}{\gamma }.$$

The steady-state distribution is Gaussian,

(12) $$p_{0}\left(x\right)\propto \text{exp}\;\left[-\frac{\gamma }{2D}(x-\beta /\gamma {)}^{2}\right].$$

Under perturbation u, the mean shifts to $x^{*}=(\beta +u)/\gamma$, which yields

(13) $$p_{u}\left(x\right)\propto \text{exp}\;\left[-\frac{\gamma }{2D}(x-(\beta +u)/\gamma {)}^{2}\right]=p_{0}\left(x\right)\cdot \text{exp}\;\left[\frac{u}{D}x+\text{const}\right].$$

Thus,

(14) $$p_{u}(x)\propto p_{0}(x)e^{ux/D}$$

showing that x is conjugate to u in the exponential family sense.

##### 1.2.2 Example 2: Bursting transcription from a two-state promoter

We consider a minimal model of transcriptional bursting with promoter switching:

(15) $$\text{OFF}\underset{k_{\text{off}}}{\overset{k_{\text{on}}}{⇌}}\text{ON},\;\text{ON}\overset{\beta }{\to }\text{mRNA}\overset{\gamma \;}{\to }\emptyset$$

Let s(t)∈{0,1} denote the promoter state and $x(t)\in N_{0}$ the mRNA count. In the *bursting regime*, we assume:

- Slow promoter switching: $k_{\text{on}},\;k_{\text{off}}\ll \gamma$
- Fast transcription when ON:β≫γ

Under these conditions, transcription occurs in bursts when the promoter switches ON briefly. The steady-state mRNA distribution is approximately exponential:

(16) $$p_{0}\left(x\right)=\frac{1}{\overset{‾}{x}}\text{exp}\;\left(-\frac{x}{\overset{‾}{x}}\right),\;x\geq 0,$$

with mean

(17) $$\overset{‾}{x}=\frac{k_{\text{on}}}{k_{\text{on}}+k_{\text{off}}}\frac{\beta }{\gamma }.$$

This can be interpreted as a bursty birth-death process:

- Bursts arrive as a Poisson process at rate $k_{\text{on}}$
- Each burst yields B~Geom(q) mRNAs with mean burst size
(18) $$b=\frac{1-q}{q}=\frac{\beta }{k_{\text{off}}}$$
- mRNAs degrade independently at rate γ

The corresponding steady-state distribution is a negative binomial:

(19) P(x)=x+r-1x(1-p)rpx,x∈N0

where

(20) $$r=\frac{k_{\text{on}}}{\gamma },\;p=\frac{b}{1+b}$$

with mean

(21) $$\overset{‾}{x}=\frac{k_{\text{on}}\cdot b}{\gamma }=\frac{k_{\text{on}}\cdot \beta }{k_{\text{off}}\cdot \gamma }$$

In the limit $b\to \infty ,k_{\text{on}}\to 0$, with $k_{\text{on}}b=\text{const}$, the negative binomial converges to the exponential distribution above.

Now consider a small perturbation u that increases $k_{\text{on}}\to k_{\text{on}}+u$. The ON-probability becomes

(22) $$\pi_{\text{ON}}(u)=\frac{k_{\text{on}}+u}{k_{\text{on}}+u+k_{\text{off}}},$$

and the perturbed mean expression is

(23) $$\overset{‾}{x}(u)=\pi_{\text{ON}}(u)\cdot \frac{\beta }{\gamma }.$$

Expanding to first order in u, we see that

(24) $$\overset{‾}{x}(u)\approx \overset{‾}{x}(0)+u\cdot \chi ,$$

with response coefficient

(25) $$\chi =\frac{\partial \overset{‾}{x}}{\partial k_{\text{on}}}=\frac{\beta }{\gamma }\cdot \frac{k_{\text{off}}}{{\left(k_{\text{on}}+k_{\text{off}}\right)}^{2}}.$$

The perturbed distribution then becomes

(26) $$p_{u}(x)=\frac{1}{\overset{‾}{x}(u)}\text{exp}\left(-\frac{x}{\overset{‾}{x}(u)}\right),$$

so to first order

(27) $$\frac{1}{\overset{‾}{x}(u)}\approx \frac{1}{\overset{‾}{x}(0)}\left(1-\frac{u\chi }{\overset{‾}{x}(0)}\right).$$

So x is conjugate to the perturbation u and

(28) $$p_{u}(x)\propto \text{exp}\left(-\frac{x}{\overset{‾}{x}(0)}\right)\cdot \text{exp}\left(\frac{u\chi x}{\overset{‾}{x}(0{)}^{2}}\right)=p_{0}(x)\cdot \text{exp}(\alpha ux)$$

with

(29) $$\alpha =\frac{\chi }{\overset{‾}{x}(0{)}^{2}}=\frac{\beta }{\gamma }\cdot \frac{k_{\text{off}}}{{\left(k_{\text{on}}+k_{\text{off}}\right)}^{2}\cdot \overset{‾}{x}(0{)}^{2}}.$$

##### 1.2.3 Example 3: Hill-type regulation with small perturbation to activator

Consider a gene x transcriptionally regulated by an activator R via a Hill function, with a small basal transcription rate β. The deterministic dynamics are

(30) $$\frac{dx}{dt}=\beta +\frac{\alpha R^{n}}{K^{n}+R^{n}}-\gamma x.$$

Here α is the maximal inducible transcription rate, K is the Hill constant, n is the Hill coefficient, and γ is the degradation rate.

At steady state, the system fluctuates around the fixed point

(31) $$x^{*}=\frac{1}{\gamma }\left(\beta +\frac{\alpha R^{n}}{K^{n}+R^{n}}\right)$$

We now consider a small perturbation u that increases the activator level: R→R+u. For small u, we expand the Hill function as

(32) $$\frac{(R+u{)}^{n}}{K^{n}+(R+u{)}^{n}}\approx \frac{R^{n}}{K^{n}+R^{n}}+\frac{d}{dR}\left(\frac{R^{n}}{K^{n}+R^{n}}\right)u$$

The derivative is

(33) $$\frac{d}{dR}\left(\frac{R^{n}}{K^{n}+R^{n}}\right)=\frac{nK^{n}R^{n-1}}{{\left(K^{n}+R^{n}\right)}^{2}},$$

so the perturbed steady-state becomes

(34) $$x^{*}\left(u\right)\approx x^{*}\left(0\right)+\frac{\alpha }{\gamma }\cdot \frac{nK^{n}R^{n-1}}{{\left(K^{n}+R^{n}\right)}^{2}}\cdot u.$$

Assuming the fluctuations around $x^{*}(u)$ are Gaussian with variance $\sigma^{2}=D/\gamma$, the steady-state distribution is

(35) $$p_{u}\left(x\right)\propto \text{exp}\;\left[-\frac{{\left(x-x^{*}\left(u\right)\right)}^{2}}{2\sigma^{2}}\right].$$

Substituting in the linear expansion for $x^{*}(u)$, we write:

(36) $$p_{u}\left(x\right)\propto \text{exp}\;\left[-\frac{{\left(x-x^{*}\left(0\right)\right)}^{2}}{2\sigma^{2}}+\frac{\left(x-x^{*}\left(0\right)\right)}{\sigma^{2}}\cdot \Delta x^{*}\right],$$

where

(37) $$\Delta x^{*}=\frac{\alpha nK^{n}{\;R}^{n-1}}{\gamma {\left(K^{n}+R^{n}\right)}^{2}}u.$$

So, finally, we have

(38) $$p_{u}\left(x\right)\propto p_{0}\left(x\right)\cdot \text{exp}\left(ux\cdot \alpha_{\text{eff}}\right),$$

with

(39) $$\alpha_{\text{eff}}=\frac{\alpha nK^{n}R^{n-1}}{\gamma \sigma^{2}{\left(K^{n}+R^{n}\right)}^{2}}.$$

#### 1.3 Perturbation-conjugate systems and the variational principle

Many biological systems are robust to small perturbations: when subjected to weak inputs u, their steady-state distribution shifts minimally from its unperturbed form. This robustness can be formalized by posing a variational problem: among all distributions p(x) that produce a given shift in the mean $\langle x\rangle =\langle x\rangle_{0}+\Delta x$, the actual response minimizes the Kullback–Leibler (KL) divergence from the original distribution $p_{0}(x)$. This is equivalent to saying the system follows the path of least surprise or minimal information cost under perturbation.

We define the optimization problem as follows,

(40) $$p^{*}\left(x\right)=\text{arg}\;\underset{p\left(x\right)}{\text{min}}\left\{D_{\text{KL}}\left(p(x)\|p_{0}(x)\right)|\int xp(x)\;dx=\langle x\rangle_{0}+\Delta x\right\},$$

where KL divergence is

(41) $$D_{\text{KL}}\left(p\|p_{0}\right)=\int dx\;p\left(x\right)\;\text{log}\frac{p(x)}{p_{0}(x)}.$$

To solve this constrained optimization, we introduce a Lagrange multiplier $u\in R^{N}$ for the mean constraint and a multiplier λ for normalization. The Lagrangian is then defined as

(42) $$ℒ\left[p\right]=\int dx\;p\left(x\right)\;\text{log}\frac{p\left(x\right)}{p_{0}\left(x\right)}-u^{⊤}\left(\int xp\left(x\right)dx-\langle x\rangle_{0}-\Delta x\right)-\lambda \left(\int p\left(x\right)dx-1\right).$$

Taking the functional derivative with respect to p(x) and setting it to zero,

(43) $$\frac{\delta ℒ}{\delta p(x)}=\text{log}\frac{p(x)}{p_{0}(x)}+1-u^{⊤}x-\lambda =0.$$

Solving this yields

(44) $$p^{*}(x)=\frac{1}{Z(u)}p_{0}(x)e^{u^{⊤}x}$$

with normalization constant

(45) $$Z\left(u\right)=\int dx\;p_{0}\left(x\right)e^{u^{⊤}x}.$$

Thus, the optimal distribution under the mean constraint is an exponential tilt of the original distribution.

This derivation shows that in any system that responds minimally to perturbations in the information-theoretic sense, the perturbed distribution takes exponential form and x is conjugate to u, guaranteeing linear response in the small-u limit.

#### 1.4 Soft modes reinforce linear response

We consider stochastic dynamics governed by the Langevin equation:

(46) $$\dot{x}=F(x)+\eta (t),\;\left\langle \eta (t)\eta^{⊤}\left(t^{\prime }\right)\right\rangle =2D\delta \left(t-t^{\prime }\right),$$

where is a constant, possibly anisotropic (i.e., non-diagonal) diffusion matrix.

Linearizing around a stable trajectory, fluctuations evolve as:

(47) $$\delta \dot{x}=J\delta x+\eta \left(t\right),\;J_{ij}={\left.\frac{\partial F_{i}}{\partial x_{j}}\right|}_{x^{*}\left(t\right)}.$$

Even if is asymmetric and is non-diagonal, the steady-state covariance matrix satisfies the continuous-time Lyapunov equation:

(18) $$J\Sigma +\Sigma J^{⊤}=-2D.$$

Now consider adding a small constant perturbation to the drift: . In the linearized system, this induces a steady-state shift:

(48) $$\left\langle \delta x\right\rangle =-J^{-1}u.$$

Suppose the dynamics are dominated by a single slow mode—a “soft mode”—such that one eigenvalue is much smaller in magnitude than the real parts of all others. Let $p_{d}$ and $q_{d}$ be the corresponding right and left eigenvectors of J, normalized such that $q_{d}^{⊤}p_{d}=1$. Then, to leading order,

(49) $$J^{-1}\approx \frac{1}{\lambda_{d}}p_{d}q_{d}^{⊤},$$

and the steady-state shift becomes

(50) $$\langle \delta x\rangle \approx \frac{1}{\lambda_{d}}p_{d}\left(q_{d}^{⊤}u\right).$$

In the same soft mode limit, the covariance matrix becomes approximately rank-1 and takes the symmetric form:

(51) $$\Sigma \approx \left(\frac{-{\tilde{D}}_{dd}}{2\lambda_{d}}\right)p_{d}p_{d}^{⊤},$$

where ${\tilde{D}}_{dd}$ is the projection of the diffusion matrix along the soft mode in the left-eigenvector (q) basis.

Crucially, both vectors point in the same direction ($p_{d}$, differing only by a scalar factor), so

(52) $$\left\langle \delta x\right\rangle =\Sigma \tilde{u},\;\tilde{u}={\left(\frac{{\tilde{D}}_{dd}}{2}\cdot \frac{p_{d}^{⊤}u}{q_{d}^{⊤}u}\right)}^{-1}u.$$

This factor can be absorbed into u in order to estimate the response from the covariance matrix. Even though $J^{-1}$ may be strongly asymmetric, the relative observable response in expression level is governed entirely by its symmetric part, which coincides (up to scale) with the covariance matrix. Thus, the linear response formula

(53) ⟨δx⟩=Σu

is valid even far from thermodynamic equilibrium, provided the system exhibits a dominant slow mode.

##### 1.4.1 Corrections from higher modes

While the leading soft-mode approximation captures the dominant structure of Σ in systems near bifurcation or criticality, we can make this approximation more precise by quantifying corrections from higher modes.

Assume J is diagonalizable, with eigen-decomposition $J=P\Lambda P^{-1}$, where $\Lambda =\text{diag}\left(\lambda_{1},\ldots ,\lambda_{N}\right)$, and P contains the right eigenvectors ${\vec{p}}_{k}$. The Lyapunov equation can be solved by transforming into the eigenbasis of J. Again, define $\tilde{\Sigma }=P^{-1}\Sigma {\left(P^{†}\right)}^{-1}$, and $\tilde{D}=P^{-1}D{\left(P^{†}\right)}^{-1}$ Then the Lyapunov equation becomes diagonal in this basis:

(54) $${\tilde{\Sigma }}_{kl}=\frac{-{\tilde{D}}_{kl}}{\lambda_{k}+{\overset{‾}{\lambda }}_{l}},$$

where $\overset{‾}{\lambda }$ denotes a complex conjugate. To return to physical coordinates, the covariance is reconstructed as:

(55) $$\Sigma =P\tilde{\Sigma }P^{†}=\sum_{k,l}\frac{-{\tilde{D}}_{kl}}{\lambda_{k}+{\overset{‾}{\lambda }}_{l}}{\vec{p}}_{k}{\vec{p}}_{l}^{†}.$$

Now assume the system has a dominant soft mode $\lambda_{d}\to 0$, with all other eigenvalues satisfying $\text{Re}\left(\lambda_{k}\right)\gg \lambda_{d}$ for k≠d. Then the leading contribution is:

(56) $$\Sigma^{\left(0\right)}=\left(\frac{-{\tilde{D}}_{dd}}{2\lambda_{d}}\right){\vec{p}}_{d}{\vec{p}}_{d}^{†}.$$

The remaining contribution comes from all (k,l)≠(d,d), forming the correction:

(57) $$\Sigma^{\left(1\right)}=\sum_{\left(k,l)\neq (d,d\right)}\frac{-{\tilde{D}}_{kl}}{\lambda_{k}+{\overset{‾}{\lambda }}_{l}}{\vec{p}}_{k}{\vec{p}}_{l}^{†}.$$

This yields the full expansion:

(58) $$\Sigma =\Sigma^{\left(0\right)}+\Sigma^{\left(1\right)}=\left(\frac{-{\tilde{D}}_{dd}}{2\lambda_{d}}\right){\vec{p}}_{d}{\vec{p}}_{d}^{†}+\sum_{\left(k,l)\neq (d,d\right)}\frac{-{\tilde{D}}_{kl}}{\lambda_{k}+{\overset{‾}{\lambda }}_{l}}{\vec{p}}_{k}{\vec{p}}_{l}^{†}.$$

The magnitude of the correction term $\Sigma^{\left(1\right)}$ is suppressed when there is a spectral gap between $\lambda_{d}$ and all other eigenvalues; the perturbation and noise project weakly onto the stiff modes ${\vec{p}}_{k}$, and the off-diagonal elements ${\tilde{D}}_{kl}$ are small, i.e., noise is nearly aligned with the eigenbasis of J.

Thus, although the soft mode approximation is leading-order accurate, this expansion systematically quantifies when and how it fails, that is, when $\lambda_{d}/\lambda_{2}\ll 1$ begins to break down.

#### 1.5 Fitting single and multi-gene response

We can judge how well correlations explain the expression difference ΔX by computing the coefficient of determination

(59) $$R^{2}=1-\frac{\left\|\Delta X-\Sigma u\right\|}{\left\|\Delta X\right\|}\leq 1.$$

As $R^{2}$ approaches 1, the correlations perfectly transport the initial vector of gene expression $\langle x\rangle_{0}$ to the final measured target $\langle x\rangle_{u}$. When $R^{2}>0.5$, for example, the initial distance between the control and perturbed gene expression has been halved.

If the cellular perturbations causing phenotypic change are known, as they are in Perturb-seq experiments, wherein it is typical for a single gene to be upregulated or knocked down per cell by CRISPRa or CRISPRi respectively, we can directly test the extent to which linear response to that single-gene perturbation effects the expected expression change. That is, for any known single-gene perturbation i, we can solve for the optimal $u_{i}$ with which to force the system towards the terminal state $\langle x\rangle_{u}$. Setting all other elements of u equal to zero, the $u_{i}$ that solves the optimization problem

(60) $$u_{i}^{*}={\text{argmin}}_{u}\|\Delta X-\Sigma u\|,\Delta X=\langle x\rangle_{u}-\langle x\rangle_{0}$$

is

(61) $$u_{i}^{*}=\left(0,\ldots ,u_{i}^{*},\ldots ,0\right),u_{i}^{*}=\frac{\Sigma_{i}^{T}\cdot \Delta X}{\Sigma_{i}^{T}\cdot \Sigma_{i}}$$

where $\Sigma_{i}$ is the ith column of Σ. We use such optimal single-gene solutions in the ‘forward’ problem below and can derive it explicitly as follows. Assuming we know which gene is perturbed we set all other elements of u to zero. Now, $\Sigma u=\Sigma_{1i}u_{i}+\ldots +\Sigma_{Ni}u_{i}\equiv \Sigma_{i}u_{i}$ where $\Sigma_{i}$ is the $i^{\text{th}}$ column of Σ. This means that the optimization in Eq. 60 reduces to

(62) $$u_{i}^{*}={\text{argmin}}_{u_{i}}{\left\|\Delta X-\Sigma_{i}u_{i}\right\|}^{2}$$

which implies

(63) $$\partial_{u_{i}^{*}}\left(\Sigma_{i}^{T}\Sigma_{i}u_{i}^{2}-2\Sigma_{i}^{T}\Delta Xu_{i}\right)=0.$$

Solving this equation for the scalar $u_{i}$ gives the desired result.

The structure of steady-state linear response dictates that we can solve for independent perturbation components $u^{*}$ by ‘decorrelating’ the observed expression change, so that

(64) $$u^{*}=\Sigma^{-1}\Delta X.$$

We interpret large elements $u_{i}$ as those driving the phenotypic change relatively independently above and beyond that encoded by the typical correlation. Relatively small elements of $u_{i}$ would then correspond to genes whose expression changes largely follow expected correlations, so that the perturbation response of that gene can largely be explained by the behavior of other genes.

### 2 Application to synthetic gene regulatory networks

#### 2.1 Random linear network

Having explained the theoretical foundations of the CIPHER framework, we now test it on a number of synthetic gene regulatory networks. We first consider the case of a linear system with random regulatory structure. That is, around a stationary fixed point centered at the origin, so that $x^{*}=0$ and δx=x, the Jacobian becomes constant and we parameterize it as -J≡A=1+ϵK, where K is an off-diagonal matrix of interactions with elements drawn from a standard normal distribution. As Robert May showed in his seminal paper on the stability of random ecosystems, A is almost surely stable when the dimension of the system N-genes satisfies the following criteria from random matrix theory: $\epsilon <\epsilon_{c}=1/\sqrt{N}$. As the network of genetic interactions becomes more and more dense, the coupling strength approaches its critical value and the smallest eigenvalue of A→0. The system bifurcates at this critical coupling, becoming globally unstable. We simulate this system using an Euler-Maryama integrator with timestep dt=0.1. For the correlations and linear response estimates in FIG 2, we use N=300 genes and integrate from the stationary state δX=0 for a total time of T=300 with a constant diffusion coefficient D=0.5 for all genes. For this system, the critical coupling is $\epsilon_{c}=1/\sqrt{N}=1/20$, and we simulate one system at $\epsilon =0.95\epsilon_{c}$, one system at criticality $\epsilon =\epsilon_{c}$ and one above the threshold $\epsilon =1.05\epsilon_{c}$. Linear response estimates are averaged over 500 independent simulation runs.

The emergence of gene teams past criticality is a direct result of the dynamics begin projected (according to center manifold theory [3]) onto the null-space of A, i.e. the direction with zero or negative eigenvalue [4]. This effective dimensionality reduction is not a result of gene teams, but the approach to and past criticality. This insight sheds light on the natural emergence of gene teams in the vicinity of a phenotypic transition; as the Jacobian becomes unstable as it would when passing through a short lived unstable fixed point, or barrier, in phase-space, mutual activation within large groups of genes and inhibition between groups becomes inevitable. This along with the fact that such gene teams have recently been posited to induce low-dimensional phenotypic structure in the epithelial to mesenchymal transition is why we study an explicit teams system below.

#### 2.2 Nonlinear Hill function network

We now move on to test CIPHER on a prototypical nonlinear dynamical gene expression system comprised of a network of hill functions [5]. Specifically, the deterministic force on the $i^{\text{th}}$ gene $x_{i}$ is $F_{i}(x)=-\gamma x_{i}+\sum_{j}G_{ij}x_{j}^{n}/\left(K^{n}+x_{j}^{n}\right)$. We apply an additional constant external force to the first gene with magnitude u and compare the true average response (from solving the stationary fixed point equations $F_{i}\left(\langle x\rangle_{u}\right)+u=0$ and $F_{i}\left(\langle x\rangle_{0}\right)=0$ to that predicted by linear response (Eq. **??**), where again J is evaluated at the unperturbed fixed point $\langle x\rangle_{0}$. For this system, we calculate the original fixed point (with u=0) and the Jacobian evaluated at that fixed point, and relate it to the new driven fixed point after perturbation. We perform these calculations using deterministic gradient flow ${\dot{x}}_{i}=F_{i}$ (towards the fixed point) for a maximum of 2000 iterations with time-step dt=0.01, stopping if the maximum change in x over an iteration is less than a fixed tolerance of 10^−12^. We consider a relatively small system N=10 to sweep over many parameters and fix $K=10,\gamma =1,G_{ij}\sim 𝒩(1,0.1)$, starting from an initial guess where all genes are at abundance $N\left\langle G_{ij}\right\rangle /\gamma =10$. When sweeping over the other parameters, we fix the Hill coefficient to n=4.

For this system, can explain the sudden jumps in the linear response error as a function of the system parameters by examining its stability. We assume a homogeneous fixed point at zero and follow what happens when we perturb around it by an amount δx, so that, averaging over the noise and assuming fixed $G_{ij}=G$,

(65) $$\delta \dot{x}=-\gamma \delta x+GN\delta x^{n}/\left(K^{n}+\delta x^{n}\right).$$

The system goes from stable to perturbation to unstable when $\delta \dot{x}=0$, so that

(66) $$\frac{G^{*}N}{\gamma^{*}}=\delta x^{n-1}/\left({\left(K^{*}\right)}^{n}+\delta x^{n}\right).$$

Solving for $G^{*},\gamma^{*}$, or $K^{*}$ yield the critical parameter values past which the system has finite average gene expression and the linear response error estimates jump.

#### 2.3 Teams network

The last synthetic system we explore here is a stochastic hybrid model of team dynamics, where genes on the same team mutually activate each other and there is inhibition between genes on different teams. Each gene has a promoter that transitions from off s=0 to on s=1, and vice versa, with rates $k_{on}$ and $k_{off}$. These rates take in input from all the other genes in the system which is modulated by a hill function. That is $k_{\text{on}/\text{off}}=1/\left(1+{\left(K/\sum_{j}B_{ij}^{\text{on}/\text{off}}x_{j}\right)}^{n}\right)$ where $B^{\text{on}/\text{off}}$ is a matrix of activating/inhibitory interactions, respectively. The force on each gene is then $F_{i}(x)=\beta s_{i}-\gamma x_{i}$ where β and γ are transcription and decay rates. This nonlinear model features collective transitions wherein all the genes on a team will cooperatively transition from off to on while the genes on the other team transition from on to off. Even in such a complex simulated system, gene expression changes upon knock-out of a single gene (setting β=0 for some gene i) are well captured by linear response. For our simulations, we fix $B_{ij}^{on}=0.3(1+𝒩(0,0.45))$, $B_{ij}^{off}=0.25(1+𝒩(0,0.45)),K=25$ and n=2. We set β=50 and γ=4 and simulate N=20 genes (10 on each team) for a total time of $T=2\times 10^{6}$ with timestep dt=0.1. We set the sparsity to S=0.7 by randomly setting elements of $B^{\text{on}/\text{off}}$ to zero with probability S. At the end of the long simulation, we calculate the gene-gene covariance and the optimal single-gene perturbation according to Eq. 61 after setting the production rate of the first gene to zero, knocking it out.

### 3 The forward problem: predicting responses from Perturb-seq

We compute the optimal single gene perturbation $u_{i}^{*}$ from Eq. 61 for each single and double-gene perturbation in each dataset, after cleaning it (see Data Processing below). We then use $u_{i}^{*}$ to calculate an $R^{2}$ value for 1) the real Σ matrix, 2) the ‘mean-field’ matrix, shuffled only over cells, not genes, and 3) the fully-’shuffled’ matrix. First we regularize the denominator in both the $R^{2}$ expression and the expression for $u_{i}^{*}$ by adding a small number 10^−8^ to avoid division by zero. In order to suppress spurious small and noisy correlations from artificially driving down $R^{2}$, in the sum what uninteresting case that the expression of a given gene does not change $\left(\Delta x_{i}=0\right)$, we only compute $R^{2}$ over genes that have nonzero response to the perturbation.

When comparing distributions and means of $R^{2}$ across datasets, we calculate p-values using Kolmogorov-Smirnoff tests and Wilcoxon signed rank t-tests (one-sided), as implemented in sciPy, respectively.

### 4 The inverse problem: predicting perturbations from correlations and response

Next, we investigated how informative the linear response estimates of the full (polygenic) perturbations in Eq. 64 are at inferring the true perturbation. For each of the 10 datasets considered, we considered ranking the genes by 1) their $u^{*}$ magnitude, 2) log-fold change, and 3) minus the log-10 p-value between control and perturbation conditions. However, since Eq. 64 only provides a point estimate of the true perturbations driving the response, we wanted to venture further to quantify the distribution of effect sizes for each gene $p\left(u_{i}\right)$ as well as the posterior inclusion probability that the effect size is truly nonzero. To do this we re-framed the linear response problem as a Bayesian linear regression [6],

(67) ΔX=Σu+ξ

where ΔX is the data, Σu is the model with Σ fixed and ξ is a random Normally distributed error term, with mean zero and variance $\sigma^{2}$. Here ξ can be thought of as modeling co-variates unaccounted for in linear response. Using Bayes’ rule, it can be shown that the posterior probability of observing u given the data is, up to a constant,

(68) $$\text{ln}\;p(u|\Delta X)=-\frac{\|\Delta X-\Sigma u{\|}^{2}}{2\sigma^{2}}+\text{ln}\;p(u)$$

where p(u) is a prior on the perturbation that we choose to be a so called Horseshoe prior [7] since it reflects the belief that the true vector of perturbations should be sparse. Since MCMC is extremely computationally intensive for large optimizations over many possible perturbations, we optimize log-likelihood lnp(u∣ΔX) over a subset of the perturbations (and corresponding rows of ΔX and block of Σ) chosen as the top-k perturbations ranked by absolute u-value according to the point estimate. We then performed Markov-Chain Monte Carlo (MCMC). As a test, we force the inclusion of the true perturbation (even if it lies outside the top-k) to see if the Bayesian estimate places it as highly probable to have non-zero effect with posterior inclusion probability (PIP) close to 1. Indeed, true perturbations that were originally ranked low can rise through the ranks during inference to become one of if not the most highly probable gene with the largest magnitude effect size of the top-k. Even when perturbations have low PIPs, their effect size distributions often have heavy tails and variances much larger than the other genes considered in the optimization, which is one reason why our chosen metric (the maximum mean-shifted spread of $p\left(u_{i}\right)$) has AUROC scores close to 1.

To maximize the posterior distribution of perturbation effects we employ the python MCMC package pyMC. We use the no u-turn sampler [8], taking 1000 MC steps after tuning for 1000 steps and accumulating samples from 8 such runs. We parameterize the sparse Horseshoe prior for each gene i as $u_{i}\sim 𝒩\left(0,\lambda_{i}^{2}\tau^{2}\right)$ where $\lambda_{i}\sim \text{Half}-\text{Cauchy}(0,1)$ and lnτ~𝒩(-4,1). We learn the noise parameter as ln σ~𝒩(-2,2). We use a conservative target acceptance rate (0.95).

For the ZINB control AUROC shown in Fig. 4G, we first fit each gene’s control expression counts to a zero-inflated negative binomial (ZINB) distribution, then randomly drew a new variance for each gene, resampled from the resulting distribution and calculated the resulting covariance. We only performed this procedure for 4 of the 10 Perturb-seq datasets considered as the others resulted in pathological individual gene distributions as they were originally too sparse to support an altered variance without pushing the mean below zero.

### 5 Eigengene analysis

Let $\Sigma =V\Lambda V^{⊤}$ be the eigendecomposition [9] of the covariance matrix with $V=\left[v_{1},\ldots ,v_{G}\right]$. Then, the projection coefficients which quantify the extent to which the response to perturbation j lies in the same direction as the eigenvectors are

(69) $$\alpha_{ij}=v_{i}^{⊤}\delta X_{j},$$

and the normalized contribution of PC i to perturbation j can be estimated as:

(70) $${\tilde{\alpha }}_{ij}^{2}=\frac{\alpha_{ij}^{2}}{\sum_{k=1}^{K}\alpha_{kj}^{2}}.$$

#### 5.1 Participation Ratio

Another measure of effective dimensionality of response to perturbation j is the participation ratio:

(71) $${\text{PR}}_{j}=\frac{{\left(\sum_{i=1}^{K}\alpha_{ij}^{2}\right)}^{2}}{\sum_{i=1}^{K}\alpha_{ij}^{4}}.$$

#### 5.2 GO enrichment analysis on principle components

We perform gene ontology (GO) enrichment [10] on the dominant axes of transcriptional variation across multiple Perturb-seq datasets. For each dataset, the we begin by computing the top 30 eigenvectors of the control covariance ordered by explained variance, which represent the primary directions of variation in control gene expression. These eigenvectors are interpreted by identifying the top 200 genes with the highest absolute loadings for each. These top genes are then submitted to g:Profiler for functional enrichment analysis against GO Biological Process (BP), Molecular Function (MF), and Reactome pathway (REAC) databases. For each eigengene, the top 5 significantly enriched terms (based on p-value) are retained. The results are aggregated into a matrix where rows represent GO or Reactome terms and columns represent eigengenes, with cell values reflecting enrichment strength (log p-value). This matrix provides a compact, interpretable summary of which biological processes are most associated with the dominant axes of gene expression variance in each dataset. All outputs—including the eigenvectors, gene rankings, enrichment results, and GO enrichment matrices—are saved for downstream analysis and visualization.

#### 5.3 Gene Contribution Fractions

Define the absolute propagated contribution to gene i:

(72) $$c_{i}=\sum_{j=1}^{G}\left|\Sigma_{ij}\right|\cdot \left|u_{j}^{*}\right|,$$

which can in general be larger than the true expression change because of possible cancellation of positive and negative correlations. The normalized global contribution vector is then

(73) $$f_{i}^{\left(\text{global}\right)}=\frac{c_{i}}{\sum_{k}c_{k}},$$

and the true perturbed target gene-specific contribution vector is

(74) $$f_{j}^{\left(\text{target}\right)}=\frac{\left|\Sigma_{gj}\right|\cdot \left|u_{j}^{*}\right|}{\sum_{k}\left|\Sigma_{gk}\right|\cdot \left|u_{k}^{*}\right|}\;\text{where}\;g=\text{perturbed}\;\text{gene}\;\text{index}.$$

#### 5.4 Effective Number of Contributing Genes (Entropy)

For a normalized distribution $f\in R^{G}$, we define the entropy-based [11] effective number of genes as:

(75) $$\text{EffSize}=\text{exp}\left(-\sum_{i=1}^{G}f_{i}\text{log}{\;f}_{i}\right).$$

#### 5.5 Self Rank

We rank the perturbed gene’s index g in its own contribution vector $f^{\text{(target)}}$, with genes sorted by descending $f_{j}$.

### 6 Data pre-processing

We apply a simple filtering procedure to the raw count matrices from the 10 studies considered here (accessed via scPerturb [12]). We begin by loading single-cell perturbation datasets in .h5ad format using Scanpy [13], computing the sparsity of the full expression matrix before filtering. We then standardize perturbation names by stripping replicate or guide suffixes (e.g., g1, g2) to extract a base perturbation label. To retain biologically meaningful genes and limit the amount of noise in the data due to extremely low expression counts, we apply an average expression filter, keeping only genes with a mean expression above a specified threshold (1 count) and ensuring that all measured perturbed genes are preserved regardless of their expression level. For the perturbations, we retain only those with at least a specified minimum number of cells (100). After filtering, we extract control cells and the selected perturbation cells.

For the single cell atlas data from CellxGene, we preprocessed all data, including reprocessing the Perturb-seq neuron datasets, as explained in the above paragraph. Datasets are preprocessed in pairs, one neuron Perturb-seq dataset and one atlas dataset, and common measured genes between the datasets in a pair are used to calculate the covariance matrices. For each cell type, we took 589 cells as this is the number of neurons in the brain atlas We found that the expression threshold of 1 count resulted in a negligible overlap in genes between datasets, so we lowered this hyperparameter value to 0.1 counts on average.

### 7 Comparison to other methods

We compared CIPHER to another linear method (MIMOSCA [14]) as well as two deep learning methods: a CPA-like autoencoder [15], and GEARS [16]. All methods were tested on RPE1 cell type data from Replogle_22a_ using the data pre-processing steps detailed above.

We implemented a simple version of the linear MIMOSCA model in Python by constructing a design matrix (cells × features) from one-hot encoded perturbation identities (except for control), and fitting a sparse Elastic Net regression. The total counts for each perturbation is appended to the perturbation labels as a covariate. For each gene we fit a regression model using the ElasticNetCV function with 5-fold cross validation and the l1 *ratio* parameter fixed to 0.5 so that the model automatically selects the L1/L2 regularization strength. After fitting the model, we predict the perturbation effect as $X1=X_{\text{design}}B$, where the columns of B are composed of the regression coefficients.

The compositional perturbation autoencoder (CPA) is a powerful machine learning method to predict perturbation responses using a latent space that treats them additively. In its original form, CPA is made for drug perturbations, so we implemented a simple version to benchmark with. Specifically, like CPA, we feed control cells and perturbation labels into neural network encoders, adding the latent variables and decoding to the perturbed expression profile (by training the decoder to reconstruct the perturbation profile corresponding to that control cell and perturbation label via a linear latent space classification loss). The model architecture includes two independent Gaussian encoders: (1) a basal encoder that maps the control transcriptome through a 2-layer MLP, and (2) a perturbation encoder that maps the perturbation one-hot vector through an identical 2-layer multi-level perceptron (MLP) to the same 64D latent space. Each encoder produces both a mean and log-variance vector, and reparameterized sampling is used to draw from the latent Gaussian. The shared latent representation is passed to a decoder consisting of a 3-layer MLP using ReLU activations and LayerNorm after each hidden layer, and a linear classifier to predict the perturbation identity. The model is trained using a composite loss consisting of (1) mean-squared error reconstruction loss between predicted and true perturbed expression; (2) cross-entropy loss over predicted perturbation identity; and (3) KL divergence for both the basal and perturbation latent Gaussians. Each loss is weighted equally. Training is conducted on CPU using the Adam optimizer with learning rate 10^−3^ for 20 epochs and batch size 10,000. Prior to training, we use the MinMaxScaler function to scale the data between 0 and 1 and we subsequently invert this transformation to produce the final generated expression profiles.

We used the GEARS Python package to train models with varying sizes and hyperparameters on our custom pre-processed data. Specifically, we trained a full GEARS model using the suggested hyperparameters: 64 encoder hidden neurons, 16 decoder hidden neurons, and 20 connections in the gene GO graph. For comparison, we also trained a reduced model with 16 encoder hidden neurons, 4 decoder hidden neurons, and 5 gene GO graph connections. All other parameters were kept at their default values, including a single network layer and a co-expression threshold of 0.4. Both models were trained for 20 epochs using a batch size of 32 for training and 128 for testing. All training was performed on A100 NVIDIA GPUs.

## Supplementary Files

This is a list of supplementary files associated with this preprint. Click to download.

- Table1KuznetsSpeckCIPHER27June2025.pdf
- FiguresSIKuznetsSpeckCIPHER5Aug2025.pdf

**Table 1: Specifications for single-cell perturbation datasets used in this study**.

**Fig. S1: Additional numerical examples on the Hill function regulatory network.** A) Relative error in linear response as a function of Hill saturation parameter K for three perturbation magnitudes.

B) Relative error in linear response as a function of decay rate γ for three perturbation magnitudes.

C) Relative error in linear response with multiple genes perturbed. For increasing Hill coefficient (left to right) we show relative error as a function of perturbation magnitude for 1–5 genes perturbed with equal magnitude. Relative error in linear response (single gene perturbed) as a function of Hill saturation parameter K for three perturbation magnitudes. Each point represents a single simulation run and lines guide the eye.

**Fig. S2: Predicting perturbation response from covariance structure in single gene perturbation** A) - F) Distributions of $R^{2}$ values over perturbations for additional datasets (labeled by dataset) for the full covariance, the mean field model and the shuffled covariance.

G) - J) Transcriptome-wide changes in gene expression over 4 genes from different datasets (lines are experimental data and points are the linear response prediction).

K) - N) Predicted vs. measured change in expression over genes (points) for the genes and datasets in G)-J).

**Fig. S3: Additional forward problem plots** A) A scatter plot of per-gene R^2^ as a function of mean control counts for a particular perturbation. All genes measured for all perturbations shown as points.

B) Distributions of R^2^ over perturbations across CRISPRi/a datasets: real covariance compared to the mean-field model.

C) $R^{2}$ distributions across all perturbations and datasets. Shuffled and mean-field histograms contain data from all perturbations and every possible perturbed gene. The true covariance $R^{2}$ histogram only includes genes that were truly perturbed.

D) $R^{2}$ histogram from linear response applied to perturb-fish imaging data: real compared to mean-field and shuffled null models.

E) R^2^ histograms for individual datasets over double perturbations. Density of R^2^ values computed with the true Σ compared to that computed with a correlation matrix from a different cell type.

F) Mean $R^{2}$ over all perturbations from datasets with combinatorial perturbations (2 or more genes perturbed). Results from the full, multi-gene optimization, the additive approximation for CIPHER, and a baseline that returns the sum of single perturbation effects are shown.

G) Density of $R^{2}$ over all perturbations using the true covariance vs. a covariance matrix from a different cell type.

H) Average $R^{2}$ for perturbations from neuron cell culture predicted by using covariance matrices from various cell types in brain, lung, and intestine single cell atlases. Error bars are calculated over atlas cell types and neuron perturbation datasets.

I) Mean $R^{2}$ vs. cell type for predicting neuron cell culture response in brain single cell atlas. Error bars are calculated over the three neuron perturbation datasets.

J) Mean $R^{2}$ vs. cell type for predicting neuron cell culture response in lung single cell atlas. Error bars are calculated over the three neuron perturbation datasets.

K) Mean $R^{2}$ vs. cell type for predicting neuron cell culture response in intestine single cell atlas. Error bars are calculated over the three neuron perturbation datasets.

**Fig. S4: Bayesian inference of true double perturbation effect sizes** A) Top two rows: examples of effect size distributions for the constituent perturbations comprising double perturbations. Third row: posterior inclusion probabilities for the double perturbations shown above (points are individual genes; true perturbed genes shown in red). Bottom row: posterior means with standard deviation error bars.

B) Distribution of rank differences of true perturbations (between the point estimate and Bayesian inference). Larger differences indicate a better final rank than initially calculated without inference.

**Fig. S5: Eigengene analysis within and across datasets.** A) Ranked eigenvalues of covariance matrices from all 10 Perturb-seq datasets.

B) Distributions of participation ratio for the remaining datasets not shown in the main figure 5A.

C) Percent that each PC falls along the direction of the response as a function of perturbation.

D) Distribution of participation ratio for all datasets and perturbations (left) and across cell type specific neuron datasets.

E) Left: Distribution of self rank over all perturbed genes. Right: the effective number of genes needed to reconstruct 99% of the response.

F) Clustered gene ontology significance heatmaps as a function of principal components over all datasets.

G) Clustered gene ontology significance heatmaps as a function of principal components over all Neuron datasets.

**Fig. S6: Comparison to other perturbation-response prediction methods.** A) Box plot of $R^{2}\left(X_{1}\right)$ against linear and deep learning methods. Median $R^{2}$ for each method is reported.

B) Histograms of $R^{2}\left(X_{1}\right)$ for each method tested.

C) Mean $R^{2}\left(X_{1}\right)$ and $R^{2}(\Delta X)$ scores for each method, shown as orange squares and blue circles, respectively. Black dashed line indicates the maximum possible score of 1.

D) Squared distance between true response and either the model prediction or average control vector. Points under the black dashed line have positive $R^{2}(\Delta X)$ and indicate that the model prediction is better than guessing the average control expression.

E) Total model parameters on a log scale.

F) Runtime in minutes to train each method for different numbers of cells. MIMOSCA was only run for the largest number of cells.

G) Memory usage in GB to train each method for different numbers of cells.

H) Total resource load (product of runtime and memory in min × GB) for the four methods.

## Data Availability

This paper analyzes existing, publicly available datasets 10/11 of which are available on the scPerturb database. All 11 datasets can be accessed through google drive (see github README for link). All code for the analyses in this manuscript has been deposited at: https://github.com/GoyalLab/CIPHER.
